# The effects of botulinum toxin A injection on the lateral pterygoid muscle in patients with a painful temporomandibular joint click: a randomized clinical trial study

**DOI:** 10.1186/s12903-022-02220-3

**Published:** 2022-05-31

**Authors:** Fahimeh Rezazadeh, Negin Esnaashari, Azita Azad, Sara Emad

**Affiliations:** 1 Department of Oral and Maxillofacial Medicine, Oral and Dental Disease Research Center, School of Dentistry, Shiraz University of Medical Sciences, Shiraz, Iran; 2 Department of Oral and Maxillofacial Medicine, Oral and Dental Disease Research Center, School of Dentistry, Yasuj University of Medical Sciences, Yasuj, Iran; 3 School of Dentistry, Shiraz University of Medical Sciences, Shiraz, Iran

**Keywords:** Lateral pterygoid muscle, Temporomandibular disorders, Botulinum toxin, Temporomandibular joint, Orofacial pain

## Abstract

**Background:**

Temporomandibular disorder (TMD) is the main cause of non-dental pain in orofacial area. The most common symptoms of TMD are joint pain, joint sound and limitation of jaw function. Botulinum toxin (BTX) injection is considered a potential treatment for TMD due to its pain-relieving properties and its ability to reduce muscle activity. Most of the studies are case series and further investigations are required to prove the efficacy of this treatment modality. Thus, in this study, we aimed to investigate the effect of BTX-A injection on the lateral pterygoid (LP) muscle and to evaluate its efficacy regarding TMD.

**Materials and methods:**

Thirty-eight patients (19 women and 19 men; mean age of 26.53 years) with painful unilateral temporomandibular joint click and LP muscle tenderness were enrolled in this study. They were divided into two groups; one received an extraoral BTX-A injection in the LP muscle, and the other received a placebo injection. Pain severity, jaw movements, click severity, and Helkimo index were recorded at the first visit, as well as one week, one month, and three months after the intervention. Data were analyzed using repeated-measures analysis of variance and *t*-tests.

**Results:**

The results showed that click severity was not significantly different between the BTX-A and placebo groups (*P* = 0.07). Pain and Helkimo index decreased significantly in the BTX group (*P* = 0.00 and *P* = 0.006, respectively); however, there was no significant difference between the two groups (*P* = 0.22 and *P* = 1, respectively). There was a significant difference in lateral movements between the groups (*P* = 0.00) but not in protrusive movement (*P* = 0.095).

**Conclusions:**

It can be concluded that although some studies have stated that BTX injection can make the click sound disappear, in this study, we did not find a significant difference between the two groups. Furthermore, our results showed that click and pain severity decreased, but the difference was not statistically significant. Therefore, further studies with a higher dosage of BTX and more participants are recommended.

*Trial registration* The local Ethics Committee of Shiraz University of Medical Sciences approved this research (IR.SUMS.REC. 01/10/2018 and IRCT20130521013406N3).

## Introduction

Temporomandibular disorders (TMDs), usually known as chronic orofacial pains, are a group of musculoskeletal diseases, which can involve masticatory muscles, temporomandibular joint (TMJ), and the associated structures [1]. TMDs are divided into two categories, i.e., intra-articular and extra-articular TMDs [2]. In more than 50% of patients, the musculoskeletal problem is the main cause of TDMs [3]. The most prevalent cause of intra-articular TMD is articular disc displacement involving the disc-condyle relationship [4].

TMD is currently considered a multifactorial disease. However, it could be caused by untreated craniofacial malocclusions, dental anomalies, psychosocial and structural dysfunctions [5, 6]. TMD is usually accompanied by anatomical, functional, and histological irregularities in musculo-articular structures; followed by various clinical symptoms, such as TMJ pain, sounds of joint, and limitation of jaw function [7]. One of the most common types of TMDs is disk displacement as internal derangement of the TMJ [8]. It may result in decreasing articular space, joint sounds (clicking, popping, or crepitation), arthritis, condylar resorption, jaw deformities, and pressure on the retrodiscal tissue, which can induce pain and dysfunction [9].

Although TMD is the main cause of non-dental pain in the orofacial area, only 5% of affected adults seek treatment [10]. The most routine non-surgical treatments are physical therapy, oral appliances, and low-level laser therapy (LLLT), which generally affect all masticatory muscles rather than a specific one [11].

Some other common conservative treatments for TMD are as follows: nonsteroidal anti-inflammatory drugs, muscle relaxants, taping, soft diet, and hot/cold compress. There are also some minimally invasive treatment alternatives such as joint lavage and intra-articular or muscular corticosteroid injection in TMJ (also known as corticosteroid infiltration), reducing pain and inflammation [12, 13]. During TMJ lavage, inflammatory mediators are washed away from the upper joint space with isotonic saline solutions [14].

As generally agreed, conservative treatments should always be the first choice for TMDs. Painful joint sound (click) is a common complaint of patients [15].

Disc displacement with reduction (DDWR) is clinically characterized by reciprocal clicking. According to a study by Ziegler et al., the routine treatment regimen consists of education, exercises, functional appliances, LLLT, and, in some cases, arthroscopy. However, clicking has not decreased significantly [16]. For treating DDWR, anterior repositioning splint was also suggested in order to maintain the normal relationship between the disc and condyle [17]. Functional appliances influence soft and hard tissue positioning. In this way, they can stimulate, or modify mandibular growth rate. However, because of poor response to treatment and potential side effects TMJ is considered difficult to treat. With regard to that, the need to novel and more treatment options should be taken into consideration [18, 19].

Botulinum toxin (BTX) is synthesized by a gram-positive, anaerobic bacterium called *Clostridium botulinum* [20, 21]. Botulinum toxin A (BTX-A) is a biologic type that paralyzes the muscle temporarily by delaying the acetylcholine production and inactivating the calcium channels in the nerve terminations [16]. According to available clinical reviews, BTX has been considered as a potential treatment for TMD due to its pain-relieving properties and its ability to reduce muscle activity [11, 22, 23]. BTX has been used extensively in treating hemifacial spasm, oromandibular dystonia, spasmodic dysphonia, and, recently, TMDs [24].

The lateral pterygoid (LP) is a two-headed muscle, which plays a major role in mastication and horizontal movements of the mandible [25]. This muscle attaches directly to the articular disc and joint capsule [26, 27]; therefore, TMDs are greatly related to anterior disk displacement [28]. Consequently, the treatment of LP muscle dysfunction is essential in TMDs followed by LP abnormalities.

Some authors have found positive effects by injecting BTX in the LP muscle, which decreased TMD symptoms; however, they are mostly case reports [8, 29–31]. Emara et al. reported a significant improvement in disc position and elimination of joint sound after BTX injection in the LP muscle [8]. In another study conducted in 2005, after BTX-A injection in LP, clicking was permanently eliminated, and the disc-condyle relationship was improved [31].

In this randomized clinical trial (RCT) study, we investigated the effect of BTX-A injection which is a widely used technique in the management of spasticity. We studied the effect of BTX-A in the LP muscle and analyzed the efficacy of this treatment modality in reducing or eliminating TMD symptoms, such as pain, clicking sound, disc placement, and mandibular movements. Thus, we compared the effect of extraoral BTX-A injection with normal saline in the LP muscle in patients with a painful click. This study adheres to CONSORT guidelines.

## Materials and methods

In this double-blind RCT study, all methods were carried out in accordance with relevant guidelines and regulations, and Informed consent was obtained from all subjects. All experimental protocols were approved by the local Ethics Committee of Shiraz University of Medical Sciences (IR.SUMS.REC. 01/10/2018 and IRCT20130521013406N3). The first date of registration of this clinical trial is: 31/12/2018.

We selected 38 patients (19 women and 19 men with a mean age of 26.53 years) referred to Shiraz Dental Faculty, Oral and Maxillofacial Disease Department; however, two men refused to continue the study. All patients had a painful unilateral TMJ click with LP muscle tenderness, while other muscles were normal. Click and TMD were diagnosed according to the research diagnostic criteria/temporomandibular disorders (RDC/TMD) criteria [3].

For examination of Lateral Pterygoid Muscle, we placed the forefinger at the area of maxillary third molar in the buccal vestibule. Then moved it in a posterior, superior and medial direction to reach maxillary tuberosity, until the lateral side of external pterygoid is palpable [32].

A panoramic radiograph (OPG) was performed before the intervention to detect possible cysts or bony abnormalities in TMJ. A complete clinical and medical examination was performed to exclude candidates suffering from jaw fractures, parafunctional habits or malocclusion, and neuromuscular, musculoskeletal, and joint disorders (bone deformities, inflammatory, septic, etc.). Patients were randomly divided into two groups (i.e., experimental [BTX, 18 patients] and control [placebo, 18 patients] groups) by block randomization [33]. All patients were older than 18 years old and informed about the planned treatment; they signed a detailed and complete written consent explaining that they might receive a Botox injection or a placebo while knowing that they all would receive the conventional pharmacotherapy prior to the study. All patients were treated with naproxen 250 mg (q 12 h) and methocarbamol 500 mg (q 8 h) for two weeks in order to alleviate the pain before any intervention.

This study was approved by the local Ethics Committee of Shiraz University of Medical Sciences (IR.SUMS.REC.1397/07/09 and IRCT20130521013406N3).

Clinical findings, such as pain severity, jaw movements, click severity, and Helkimo index (for TMD evaluation), were recorded at the first visit, as well as one week, one month, and three months after the intervention.

TMJ pain severity was evaluated according to the visual analog scale (VAS) [34]. We also measured maximal interincisal opening (MIO), range of lateral movement, and protrusion of jaw by a calibrated coulis (INSIZE, China), recorded in millimeters.

Click severity was assessed according to the following criterion: 0 = no sound, 1 = little sound (could not be heard without a stethoscope), 2 = loud sound (could be heard without a stethoscope). Patients in the experimental group received BTX-A extraoral injection in the LP muscle on the clicking side.

**Fig. 1:**
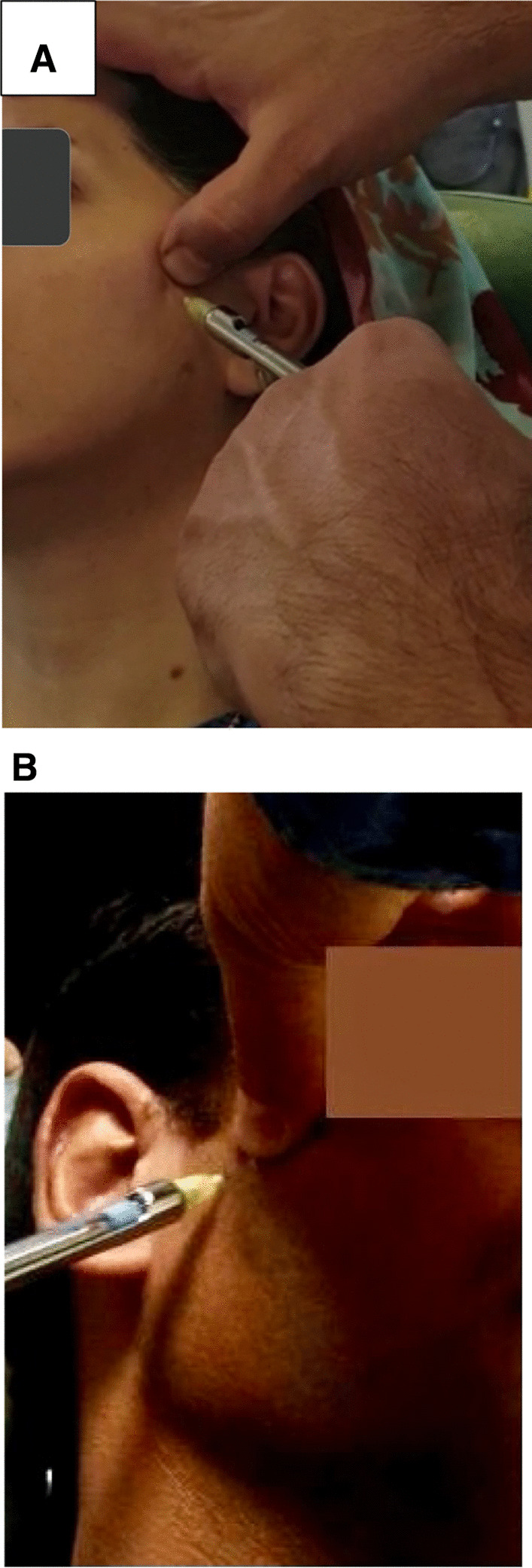
Site of injections. **A** BTX injection. **B** Normal saline injection

The BTX-A vial was reconstituted by 2 mL of 0.9% normal saline to prepare a 15 U/0.1 mL solution (Dysport 300 U powder for injection) (*C. botulinum* type A neurotoxin complex) (Dysport, Ipsen, Mulholland, UK); 0.1 mL of this solution containing 15 U BTX-A was used for injection with a dental syringe. Injection was carried out by an oral and maxillofacial specialist with the same method as Fu et al. [35]. Accordingly, the syringe was injected extraorally into the LP muscle through the skin in the coronoid notch area (Fig. 1) [36]. It was inserted anterior to the condylar neck with a 45° angle, 1 cm below the central zygomatic arch and 0.5–1 cm anterior to the condyle of the mandible to reach the correct position [37]. Aspiration was carried out to avoid unintentional intravascular injection since the muscle is surrounded by the pterygoid venous plexus.

All patients were followed up one week, one month, and three months after the intervention, and measurements were recorded again. For the control group (placebo), we injected normal saline with the same volume and in the same area.

The sample size was designed based on the statistical analysis. Data were analyzed using SPSS 22 (SPSS Inc., Chicago, Ill., USA), applying mean ± SD and frequency (%). The paired *t* test was applied to compare all data (such as age, pain, and Helkimo index) prior to the study. The *t* test was employed in order to compare the mean differences in the Helkimo index and VAS. Repeated measures analysis of variance (ANOVA) was used to assess changes in pain scores between the study groups. In all the analyses, results were considered statistically significant when the *P* value was equal to or less than 0.05.

## Results

Out of 38 patients, two men refused to continue the study; therefore, 18 candidates in the BTX group and 18 in the placebo group completed all treatment sessions. The mean age of patients was 26.53 years old (28.28 $\pm \hspace{0.17em}7.9$ in the BTX group and 24.7$8\hspace{0.17em}\pm \hspace{0.17em}$4.5 in the placebo group). Mean age, pain severity (VAS), and Helkimo index in the two groups were not significantly different according to the *t* test prior to the intervention (*P* = 0.11, *P* = 0.13, and *P* = 0.37, respectively). Regarding the chi-square test, both groups were similar in gender distribution (*P* = 0.5; Table 1).

**Table 1:** Demographic information of the experimental and control groups

Group	Number	Female	Male	Mean age (year)
Experimental (BTX)	18	11	7	28.78
Control (placebo)	18	8	10	24.78

In both BTX and placebo groups, a significant decrease was found in click severity considering the follow-up visit one month after injection (*P* = 0.05 and *P* = 0.001, respectively). However, in month 3, the click severity decreased in the BTX group (*P* = 0.06), and, in the placebo group, it increased (*P* = 0.137); however, the difference was not statistically significant (*P* = 0.07; Table 2).

**Table 2:** Means of variables in the BTX-A and placebo (normal saline) groups in different follow-up periods

Group	Measurement						
	Time	Click*	VAS	Maximum opening (mm)	Lateral movement (mm)	Protrusion movement (mm)	Helkimo index
BTX-A	Baseline	2.00	4.72	43.45	8.28	7.46	7.77
	One week		2.11	43.89	8.15	7.58	
	One month	1.05	1.78	43.86	8.15	8.21	3.5
	Three months	1.07	2.00	43.66	8.54	7.49	3.38
Placebo	Baseline	2.00	3.50	49.82	9.01	6.83	6.56
	One week		1.78	47.27	8.54	6.40	
	One month	1.11	1.72	46.63	8.08	6.26	2.86
	Three months	1.52	1.89	46.11	7.55	5.98	4.61

*Click: 0 = no sound, 1 = little sound (could not be heard without a stethoscope) and 2 = loud sound (could be heard without a stethoscope)

**Fig. 2:**
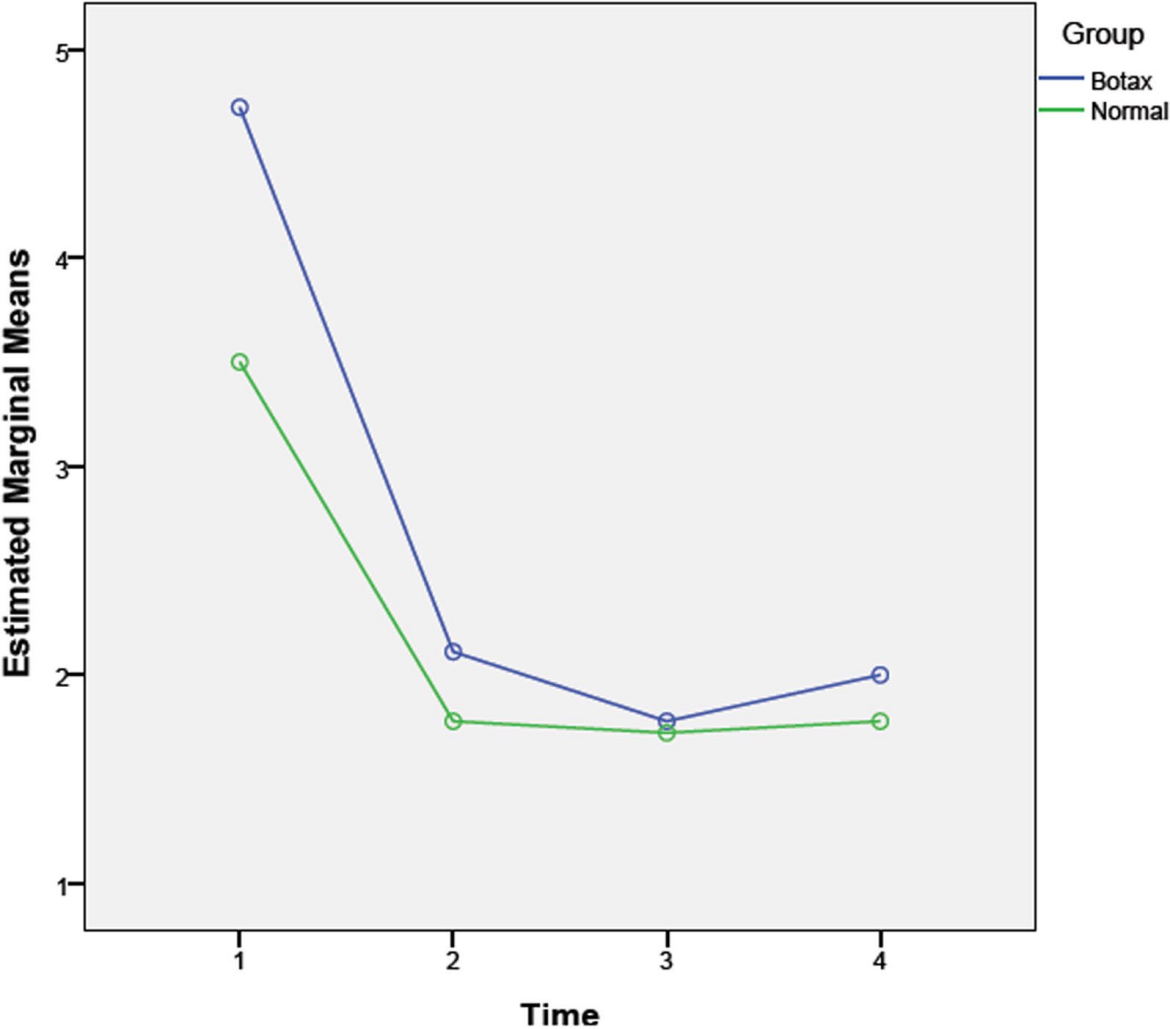
Comparing pain severity (VAS) between the BTX and control (normal saline) groups

The effect of BTX injection on pain severity was evaluated by a repeated measures-ANOVA model. The pain decreased significantly after one week and lasted for three months after BTX injection (*P* = 0.00; Fig. 2).

Comparing BTX and placebo groups, there was no significant difference in pain severity during all follow-up periods (*P* = 0.22). Hence, both groups had a similar trend in pain relief.

According to the *t* test, the Helkimo index significantly decreased after one and three months in the BTX group (*P* = 0.000 and *P* = 0.000, respectively); however, there was no significant difference between these two follow-up periods based on the Helkimo index (*P* = 0.99). In the placebo group, the Helkimo index significantly decreased after one month (*P* = 0.000); however, after three months, the Helkimo index significantly increased compared to the first month (*P* = 0.002). Besides, there was no significant difference between the BTX and placebo groups based on the Helkimo index at the end of three months (*P* = 0.18).

**Fig. 3:**
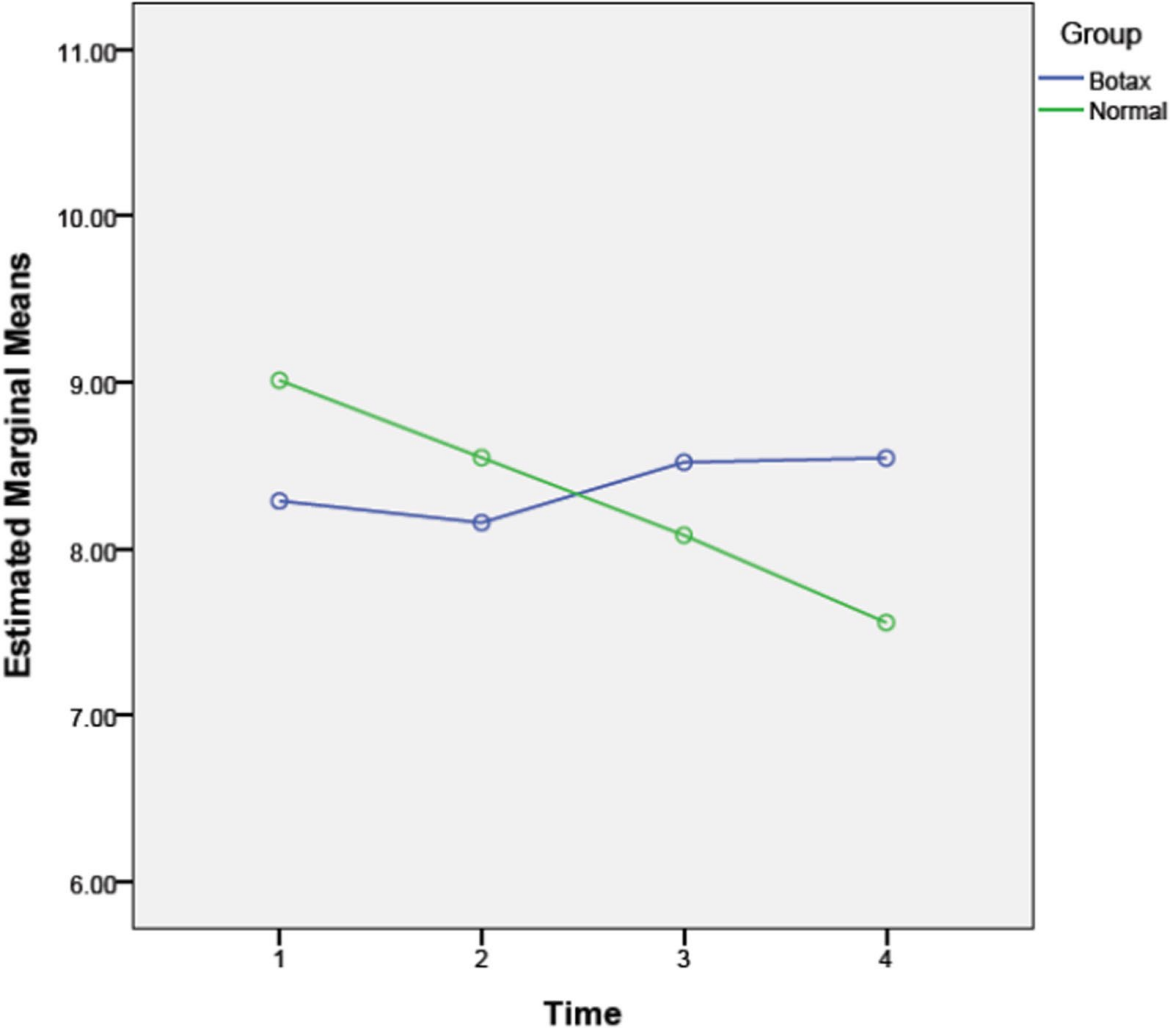
Comparing lateral movement between the BTX and control (normal saline) groups

When assessing jaw movements in the BTX group, the maximum opening was not significantly changed in follow-up visits (*P* = 0.17), and there was no difference between the placebo and BTX groups (*P* = 0.08). There was a significant difference in lateral movements between the two groups (*P* = 0.00). Lateral movement in the BTX group gradually increased during three months, but it was not statistically significant (*P* = 0.59). In contrast, in the placebo group, the mean of lateral movement decreased by the time (especially after three months compared to the baseline, *P* = 0.02; Fig. 3).

**Fig. 4:**
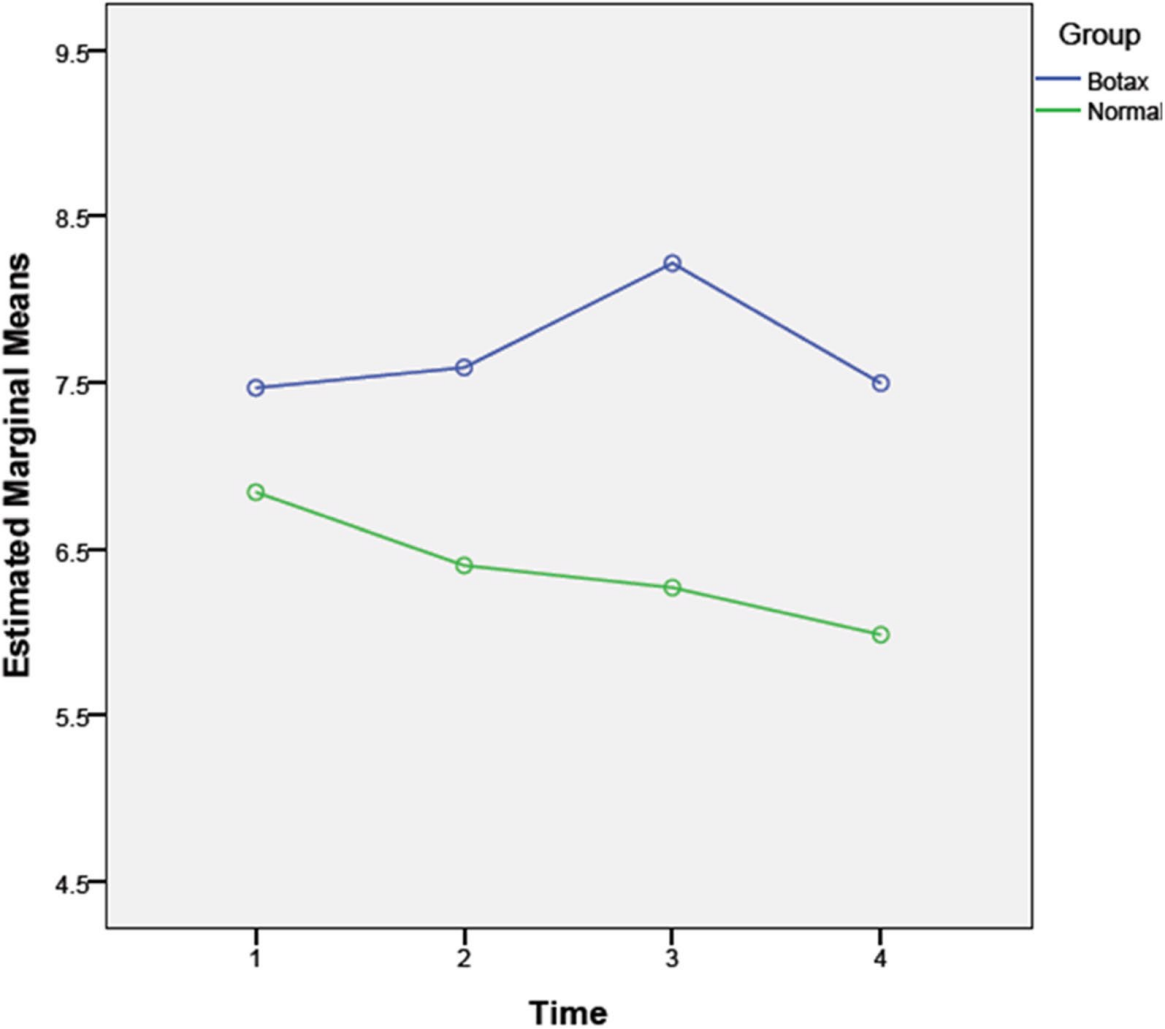
Comparing protrusion movement between the BTX and control (normal saline) groups

There was no significant difference in lateral movement between the two groups in different periods (one week [*P* = 0.12], one month [*P* = 0.52], and three months [*P* = 0.17]). There was a significant difference in the changes of protrusion movement between the two groups (*P* = 0.00). Protrusion movement in the BTX group gradually increased, especially during the first month but returned to the baseline after three months; however, these changes were not statistically significant (*P* = 0.23). In the placebo group, protrusion movement gradually decreased during three months, but it was not significant (*P* = 0.66; Fig. 4).

Generally, there was no significant difference in protrusion movement between groups during follow-up periods (*P* = 0.095).

## Discussion

The results showed that although BTX-A decreased click severity after three months compared to the placebo group, there was no significant difference in resolving click between the two groups. It was also seen that both BTX and normal saline injections reduced the click sound after one month. However, in month 3, there was a decrease in click severity only in the BTX group, although not significant; which can indicate the effectiveness of BTX in our study.

It is also notable that maximum opening and lateral and protrusive movements increased in the BTX-A group when comparing the results in month 3 with the baseline; however, the differences were not statistically significant (Table 2).

To our knowledge, there are only a few similar studies which have evaluated the effects of BTX-A injection on click severity and these studies were mostly limited to case series [30]. The present study was designed according to the promising preliminary results of Bakke et al. [9] and Emara et al. [8].

They reported the successful use of BTX-A injection as a treatment for TMJ clicking; however, these studies were case reports and case series. On the other hand, our study was designed as an RCT study and compared the effects of BTX-A injection on TMD with normal saline.

The difference between our results and other similar studies [8, 9] could be due to several reasons; these studies are case series without placebo groups. Also, it could be due to different methods, BTX dosages, and frequencies of injections. These studies used an intraoral route for access to the LP muscle, but we used an extraoral method based on Fu et al.’s study [35]. The volume of injection was also lower than other studies due to the probable risk of hemorrhage as a result of proximity to the maxillary artery and pterygoid venous plexus. The other cause of this insignificant result might be unilateral injection. It should also be mentioned that the mean age of the BTX-A group was four years younger than the control group (Table 1); thus, it can be expected that in the control group, the problem diminished as self-limit compared to the BTX-A group. Above all, a larger sample size can result in more specific results.

Based on the present study, pain severity (VAS) was significantly reduced after one week following the injection. The mean VAS in the BTX group was lower than in the placebo group, but it was not significantly different. Normal saline may wash joint space, decrease inflammatory mediators, and act as joint lavage. Similar to our results, Emara et al., Bakke et al., and Kurtoglu et al. [8, 9] reported that pain decreased and psychological status improved during the time.

On the other hand, psychological effects might affect our results (the so-called placebo effect). This may confirm that despite decreasing click sound during the first week, it returns after one month in the placebo group. Overall, patients were satisfied with the treatment, especially during the first month. It should also be noted that all patients received medicine (i.e., non-steroidal anti-inflammatory drugs [NSAIDs] and muscle relaxants) for two weeks before the intervention and were trained to follow instructions, such as eating soft foods, chewing bilaterally, using warm packs, etc.); hence, this can itself have a role in improving symptoms in both groups.

In von Lindern’s study, 200 U BTX was used for all masticatory muscles (such as masseter, temporalis, and LP) for the treatment of painful hyperactivity, parafunctions, and hypermobility of the jaw; results were satisfactory regarding pain reduction [38]. Muscles act as a team, and relaxing them can significantly reduce pain. Also, Karacalar et al. [23] used BTX in both medial and LP muscles, resulted in satisfactory outcomes; the use of these two muscles as one unit might be better to release pain faster, though we paralyzed only the LP muscle because of its role in anterior disk displacement (click) due to its attachment to the disc.

The dosage of BTX for injection varied in different studies, which depends on muscle bulk and the site of injection. For masticatory muscles (temporalis and masseter), the recommended dose of BTX for each muscle ranges from 40 to 60 U, each at several injection points [39]. Since LP is a small muscle (located deeply and adjacent to several vital structures) and may be affected by seepage, it requires a lower dose, and the injection should be made at a single point. In some studies [8, 9], 35 U was injected intraoral, and other studies used 50 U in the LP muscle, but this was accompanied by a higher percentage of side effects such as dysphagia [40]. Accordingly, we used 15 U of BTX for injection in the LP muscle.

We used an extraoral approach like Fu et al. and Ziegler et al. in our study [16, 35] because extraoral injection was more comfortable than intraoral injection. Therefore, 15 U of BTX was injected into the LP muscle, and patients were followed up as mentioned in the method section. Based on our results, it seems that the intraoral approach may have better results in paralysis of LP.

Similar to other studies, maximum jaw opening did not change [8, 9], but the mean of lateral movement and protrusion increased gradually in the BTX group. LP relaxation is a reason that patients can move their jaws more comfortably without pain, although it was not statistically different compared to the placebo group.

The initial diagnosis of click was confirmed by clinical examination and complaints of patients based on the American Academy of Orofacial pain criteria. One of the benefits of the present study was the use of different measurements such as the Helkimo index, VAS, and all of the movements separately for better evaluation. The Helkimo index was measured before and after treatment, and it was a positive point because previous studies did not measure it. This index was used to rule out the psychological effects [41]. TMJ series radiography also was performed, and pathologic problems were excluded from the study.

This study has some limitations; it is better to evaluate the disk position before and after treatment with magnetic resonance imaging (MRI) because it shows the disk position better and can detect it more properly. Electromyography is a useful device assuring that the needle enters into the muscle properly and not into space. However, in this study, like alveolar nerve block injection, we used anatomical points.

## Conclusion

The injection of BTX-A into masticatory muscles for the treatment of TMD is routinely used, but there are not enough studies on click specifically; hence, we performed this method as RCT. It is also assumed that BTX could be considered as an appropriate substitute for corticosteroid injections in treating TMD, especially in patients who are contraindicated to receive corticosteroids due to some special conditions, such as osteoporosis, uncontrolled hyperglycemia, diabetes mellitus, etc. Our results showed that click and VAS decreased after BTX injection, but the difference was not statistically significant compared to the control group. Therefore, further studies with higher dosage of BTX and more participants seem to be helpful. It is well known that MRI is the best method to evaluate TMJ status; however, in our study, due to financial and other limitations, we could not use it. Therefore, we recommend further studies to use MRI or cone beam computed tomography (CBCT) as more accurate diagnostic methods to assess TMJ.

## Data Availability

The datasets used and analyzed during the current study are available from the corresponding author on reasonable request.

## Abbreviations

BTX-A: Botulinum toxin-A
LP: Lateral pterygoid
TMD: Temporomandibular disorder
BTX: Botulinum toxin
TMJ: Temporomandibular joint
LLLT: Low-level laser therapy
DDWR: Disc displacement with reduction
RCT: Randomized clinical trial
VAS: Visual analogue scale
MIO: Maximal interincisal opening
mm: Millimeters
MRI: Magnetic resonance imaging
CBCT: Cone beam computed tomography
